# Avoidance of parasitic fungi and nematode threats by *Tribolium castaneum*

**DOI:** 10.1093/beheco/arag071

**Published:** 2026-06-10

**Authors:** Timothy R Smith, Janet Koprivnikar

**Affiliations:** Department of Chemistry and Biology, Toronto Metropolitan University, 350 Victoria Street, Toronto, ON M5B 2K3, Canada; Department of Chemistry and Biology, Toronto Metropolitan University, 350 Victoria Street, Toronto, ON M5B 2K3, Canada

**Keywords:** behavior, risk, parasite, pathogen, threat, assessment

## Abstract

Just as for predators, many animals have developed a suite of defenses to counter the ubiquitous threat posed by parasites. Behavioral avoidance is a key strategy to reduce the likelihood of encountering situations with a relatively high degree of risk. However, because such avoidance likely incurs costs (ie nonconsumptive effects), individuals should engage in risk assessment to gauge their threat of parasitism based on relevant cues. Combinations of cues may signal particularly risky situations, especially as animals often face concurrent natural enemies. In the first study to consider host choice in the presence of different parasites, we investigated behavioral avoidance in red flour beetles (*Tribolium castaneum*) given various foraging options involving 2 entomopathogens, the fungus *Beauveria bassiana* and the nematode *Steinernema carpocapsae*. Combinations included single and dual parasite threats, as well as whether parasites were represented by the presence of infectious stages and/or conspecific cadavers that were euthanized or killed by infection. Beetles generally did not exhibit behavioral avoidance of either parasite or the parasite-killed conspecifics, but rather, tended to be attracted to the latter if nematode infected. They also did not avoid conditions with mixed cues related to the threat of infection, suggesting that they do not gauge or respond to parasitism risk in this way. Our findings have implications for understanding avoidance as an antiparasite behavior across different host taxa, but also for the use of biocontrols, such as fungi and nematodes, for pest insects.

## Teaser

Choosing to avoid contact with a predator over a parasite seems relatively simple but what about the risk posed by two different parasites? In the first study to consider threat assessment of this type, we found that red flour beetles did not prioritize avoidance of either a parasitic fungus or roundworm, nor did they show a stronger response to multiple threat cues. As important pests of stored foods, this has implications for biocontrol measures.

## Introduction

Natural enemies, such as predators, can have substantial effects on individual prey and prey populations through mortality, ie by consumptive effects (CEs), as seen with the classic lynx and snowshoe hare cycles (O'Donoghue et al. 1997). However, predators can also have nonconsumptive effects (NCEs) on prey as the latter may undergo various trait changes in response to predation risk (Peckarsky et al. 2008; Sheriff and Thaler 2014; Peacor et al. 2020; Sheriff et al. 2020). This includes behavioral changes, such as spatiotemporal avoidance of predators (eg Dodson 1988). Importantly, predator avoidance can lead to trade-offs with other activities, such as optimal foraging or looking for shelter (see review by Peckarsky et al. 2008). Because of this potential for NCEs stemming from avoidance, prey should be able to respond appropriately to changes in cues that signal predation risk (see reviews by Wirsing et al. 2021; Crane et al. 2024). In other words, they should engage in risk assessment and exhibit predator avoidance only when the threat is relatively high to not incur the cost(s) of such behaviors when unnecessary (eg Ferrari et al. 2006). Conspecific cues are also important for appropriately assessing predation threat (Peacor 2003), such as per capita risk based on density (Van Buskirk et al. 2011).

While predators are important natural enemies that can exert both CEs and NCEs, parasites and pathogens (hereafter parasites) are also an ubiquitous threat to most organisms and have many similarities to predators as natural enemies in an ecological context, such as regulating the populations of their victims (Raffel et al. 2008). The CEs of parasites can manifest in many ways, from higher energetic expenditures and mortality in hosts to fitness reductions (eg Agnew et al. 2000; Hicks et al. 2018). This said, their capacity for NCEs on potential hosts has been considered only relatively recently (eg Buck et al. 2018; Weinstein et al. 2018a; Koprivnikar et al. 2021a, 2021b; Liang and Luong 2024), largely in the context of antiparasite behaviors, such as avoidance.

Avoidance is a common behavioral strategy in response to the risk of parasitism. Similar to avoiding predators, potential hosts can show spatial and/or temporal changes in some aspects of their behavior to limit their exposure to situations with high infection risk. This can involve avoidance of parasite infectious stages found in the environment, as well as infected conspecifics or proxy cues associated with infection threat, such as feces (Buck et al. 2018). These parasite avoidance behaviors have been described as “the ecology of disgust” and the “ecology of fear” depending on the mechanisms involved (see review by Love et al. 2024). Parasite avoidance has been reported in a wide range of animals (see reviews by Behringer et al. 2018; Hart and Hart 2018; Sarabian et al. 2018; Vale et al. 2018; Gibson and Amoroso 2022). For instance, potential hosts will forego foraging in areas where there is a risk of infection (eg Koprivnikar and Penalva 2015; Coulson et al. 2018). Just as for predation risk, conspecific cues are also important in avoiding the threat of parasite infection (eg Poulin et al. 1999; Daversa et al. 2021a).

However, like predator avoidance, behaviors to reduce the risk of acquiring parasite infections can be costly such that parasites also exert NCEs. Most of these involve the loss of opportunity as activities placing hosts at risk of parasite contact are also necessary for their survival and fitness, eg foraging and searching for mates or shelter (Weinstein et al. 2018a). As an example, deer reduce foraging in areas highly infested with ticks, sacrificing overall feeding opportunity to avoid infection (Allan et al. 2010). Given these trade-offs, hosts should engage in parasite threat assessment, as for predator risk, tailoring their avoidance behaviors as necessary (eg Weinstein et al. 2018b). Some species also demonstrate a fine-tuned ability to assess infection threat, such as fruit flies (*Drosophila nigrospiracula*) exhibiting avoidance of parasitic but not free-living mites (MacLeod and Luong 2024).

Importantly, many organisms face concurrent threats by different natural enemies, with implications for avoidance behaviors depending on whether these behaviors are compatible or in conflict. Studies considering host responses to the simultaneous threat of both predators and parasites have reported several outcomes that may be difficult to predict without a food web context (Doherty and Ruehle 2020). For instance, *Rana pipiens* tadpoles prefer to forage in the presence of parasite cues over those of odonate predators (Koprivnikar and Penalva 2015)—a commonly reported outcome as predation is a greater threat to fitness than infection (Daversa et al. 2021b). However, this outcome is not universal (eg Baker and Smith 1997). It is important to note that no studies to date have considered parasite avoidance when potential hosts are confronted with a concurrent threat posed by 2 different parasite types—this is a major gap in our knowledge given that this situation is likely very common in nature.

While parasite avoidance has been relatively well studied for many vertebrates, insects also exhibit such behaviors (eg Zélé et al. 2019; reviewed by De Roode and Lefèvre 2012). The threat posed by insect-infecting parasites (ie entomopathogens) is particularly serious as many are lethal and must kill their host to complete their life cycle. This includes entomopathogenic nematodes (EPNs), such as *Steinernema carpocapsae* (SC), and fungi, such as *Beauveria bassiana* (BB), which are natural threats to many insects, infecting a range of species on every continent except Antarctica (Meyling and Eilenberg 2007; Bhat et al. 2020). Many entomopathogenic fungi and nematodes share important similarities that include penetrating their host, killing it, and then colonizing its body before new infectious stages emerge from the cadaver (Clarkson and Charnley 1996; Dillman et al. 2012; Zhang et al. 2025).

Such obligate host killers make parasite avoidance a critical frontline defense. For instance, ants of various species (eg *Polyrhachis dives*) have many effective strategies against fungal infections (Heinze and Walter 2010; Tranter et al. 2015) and female parasitoid wasps (*Mastrus ridibundus* and *Liotryphon caudatus*) can seemingly detect potential hosts infected with SC and avoiding ovipositing in them (Lacey et al. 2003). Parasite avoidance behavior in insects can also be context dependent. For instance, larval mealworm beetles (*Tenebrio molitor*) avoid wheat kernels contaminated with various types of toxin-producing plant fungi or BB depending on their associated mortality and weight gain (Guo et al. 2014), and larval ambrosia beetles (*Xyleborinus saxesenii*) avoid BB while adult females do not, presumably because the larvae are more vulnerable to infection (Diehl et al. 2023).

Central to tailoring avoidance to the risk posed by a given parasite at any time or place is the ability of potential hosts to detect and assess relevant cues. As noted above, risk-signaling cues are not only generated by parasite infectious stages within the environment, but cues can also emanate from conspecifics, even dead hosts, especially as infectious stages often occur in great numbers around host cadavers. Conspecific cadavers can also signal general danger and have a repellent effect, as seen with the red flour beetle *Tribolium castaneum* (Trematerra et al. 1996). Red flour beetles secrete 1-pentadecene, which triggers avoidance behavior at high concentrations (Suzuki et al. 1975; Đukić et al. 2021), but other compounds could be involved, including fatty acids signaling death in other insects (Sun et al. 2013).

Notably, insects may respond to highly specific cues from cadavers in the context of lethal parasite threat, as seen in the cucumber beetle *Acalymma vittatum*, which specifically avoids dead nematode-infected conspecifics, but not uninfected cadavers (Grunseich et al. 2021). As noted above, insect avoidance of both parasitic fungi and nematodes has been observed and likely involves host detection of volatile organic compounds (VOCs). For instance, fungal avoidance by beetles (*Coptotermes formosanus)* and termites (*Rhynchophorus ferrugineus*) may be driven by VOCs emitted by the spores/conidia released from dead hosts (Hussain et al. 2010; Jalinas et al. 2022), and EPNs can also release cues triggering avoidance by *A. vittatum* beetles (Grunseich et al. 2021). However, infection may alter the VOC profiles of conspecific cadavers, meaning that the chemicals associated with infected cadavers could be distinct from those of conspecific cadavers killed by other means (Grunseich et al. 2021; Naundrup et al. 2022).

Examining fungal and nematode avoidance in insects will help us to better understand antiparasite behaviors in this extremely diverse and important taxon. It is also essential to study the extent to which potential hosts assess parasite risk before engaging in avoidance, especially the possible cues involved. As noted earlier, how potential hosts respond to the simultaneous threat posed by different parasites is completely unknown. Such questions are particularly relevant when considering control strategies for insect pests, such as *T. castaneum*, given the range of stored foods upon which they feed, including flour, grains, and beans (Padin et al. 2002; Jung et al. 2020). Flour beetles can also serve as the intermediate host of the tapeworm *Hymenolepis diminuta*, possibly infecting humans if they consume infected beetles from contaminated food sources (Makki et al. 2017). This makes flour beetles a common target for bioinsecticides, including various fungi and nematodes (Padin et al. 2002; Ramos-Rodríguez et al. 2006).

This study explores red flour beetle avoidance of the risk posed by the fungus BB and the nematode SC. We considered beetle avoidance of areas containing only BB or SC infectious stages (single or concurrent), but also beetle avoidance of conspecifics that were either euthanized or parasite killed—this allowed us to examine the potential contributions of host- and parasite-generated cues to threat assessment through a series of complementary choice tests. Our overall hypothesis was that beetles would avoid the higher risk/threat option in each choice test, as represented by the presence of a single threat (or the more lethal threat), or the presence of multiple threat cues over single-threat cues. As such, we predicted beetle avoidance of BB or SC over no threat, avoidance of dual parasite threat over single parasite threat, and avoidance of parasite-killed conspecifics over euthanized conspecifics (ECs) in various combinations.

## Materials and methods

### Experimental design

To study insect avoidance of 2 different entomopathogens, and what cues might influence such behaviors, we tested *T. castaneum* (red flour beetles) using choice arenas with 2 chambers of flour (a food attractant) to which we assigned different levels of parasite threat. These chambers held cues meant to signal single or dual parasite threat from BB or SC infectious stages associated with the flour medium (the parasite-only cluster of choice tests in Table 1), as well as various combinations of each parasite's infectious stage and conspecific cadavers that had been euthanized or killed by infection (the BB mixed cue tests and SC mixed cue tests in Table 1). BB infects insects through spores (conidia) that penetrate the host cuticle and then proliferate throughout the hemocoel, causing host death before the fungus emerges from the cadaver and grows spore-producing structures on the cadaver's surface (Feng et al. 1994). SC must also kill its host to complete its life cycle. Insects become infected when they contact the third-stage infective juveniles (IJs) of SC that gain host entry through natural openings. Once inside, these juveniles release endosymbiotic bacteria that kill the insect, allowing the parasite to molt and become reproductive adults, resulting in eggs that hatch and develop into new third-stage IJs that emerge from the insect's cadaver and search for new hosts (Burnell and Stock 2000).

**Table 1:** Separate choice tests (indicated by letter) by cluster corresponding to single or mixed parasite threat cues represented by the infectious stages of the fungus BB and the nematode SC, as well as euthanized or parasite-killed cadavers, indicating the high and low threat conditions for each choice test, as well as its statistical output.

Choice test	Choice 1 (high threat)	Choice 2	Statistical output
***Parasite only***			
**A**	BB	No threat	F_1,22_ = 0.335, *P* = 0.569
**B**	SC	No threat	F_1,24_ = 0.288, *P* = 0.596
**C**	**BB**	SC	F_1,20_ = 6.375, ***P*** **=** **0.02**
**D**	BB + SC	SC	F_1,18_ = 0.100, *P* = 0.755
**E**	BB + SC	BB	F_1,22_ = 1.247, *P* = 0.276
**K**	**Euthanized cadaver**	No threat	F_1,28_ = 8.081, ***P*** **=** **0.008**
***SC mixed cues***			
**F**	SC-killed cadaver	**No threat**	F_1,26_ = 8.994, ***P*** **=** **0.006**
**G**	SC + euthanized cadaver	SC	F_1,26_ = 1.239, *P* = 0.275
**H**	SC + euthanized cadaver	Euthanized cadaver	F_1,28_ = 0.245, *P* = 0.625
**I**	SC + SC-killed cadaver	**SC** **+** **euthanized cadaver**	F_1,30_ = 3.914, ***P*** **=** **0.057**
**J**	**SC-killed cadaver**	Euthanized cadaver	F_1,30_ = 6.340, ***P*** **=** **0.017**
***BB mixed cues***			
**L**	BB-killed cadaver	No threat	F_1,28_ = 3.834, *P* = 0.06
**M**	BB + euthanized cadaver	BB	F_1,30_ = 0.301, *P* = 0.587
**N**	BB + euthanized cadaver	Euthanized cadaver	F_1,30_ = 0.001, *P* = 0.978
**O**	BB + BB-killed cadaver	BB + euthanized cadaver	F_1,22_ = 0.015, *P* = 0.902
***P***	BB-killed cadaver	Euthanized cadaver	F_1,24_ = 0.934, *P* = 0.343

Note that *P* values with statistical significance are indicated by bold font after applying a Benjamini–Hochberg correction for the choice tests in each cluster using a false discovery rate of 0.25. For significantly different choice tests, the avoided choice is bolded.

### Beetle and parasite culture

*T. castaneum* (Carolina Biological) were cultured in groups of approximately 50 beetles in 1 L glass jars that contained 3 cups of flour media. The latter was a 4:4:1 ratio of whole wheat flour, white flour, and baker's yeast, respectively, as recommended by the supplier. The beetles were stored in darkness and kept at ∼20 °C with relative humidity at 48%. All beetles used in the study were haphazardly selected adults (ie not sorted by sex) that were <2 wk postemergence from their pupal stage for each choice test.

*B. bassiana* (ATCC74040, obtained from the American Type Culture Collection, ATCC) was chosen to evaluate behavioral avoidance by *T. castaneum* because it has lethal effects on its insect hosts (Oreste et al. 2012), including *T. castaneum*, which we also verified through our own pilot tests (see below). We cultured BB using established approaches (eg Ortiz-Urquiza et al. 2010; Smith and Koprivnikar 2024) and maintained the cultures on plates of Sabouraud Dextrose Agar (SDA) in an incubator (Yamato 37 L benchtop, model 1C-103C) at 25 °C. BB isolates used in the behavioral experiments or for cadaver production (see below) were maintained for 14 d before conidia were harvested in a 0.05% Tween 80 solution.

We used IJs of SC for this study (Capsanem obtained from Koppert Biological Systems). To quantify SC lethality to *T. castaneum,* we conducted pilot trials in which Petri dishes lined with 90 mm grade 1 Whatman filter papers were inoculated with the same concentration of parasite used in the behavioral trials (10^7^ conidia/mL for BB and 1 IJ/µL for SC). The pilot infection trials contained 3 conditions, each involving 10 beetles: exposure to BB, exposure to SC, and a negative control inoculated with distilled water (DW). Once the filter paper had dried, individual beetles were placed in Petri dishes and stored at 25 °C. Their survival was checked daily for 5 d, and then twice weekly following that.

### Cadaver production

To investigate possible cues used by red flour beetles to avoid conditions representing different degrees of parasite risk, cadavers were used in several of the choice tests (tests F to P in Table 1). However, we wanted to distinguish between conspecifics that were killed by either parasite or which died through other means as these could provide additional signals related to infection risk beyond any generated by the parasites themselves. While parasite-killed cadavers would contain the infectious stages of the parasite that killed them, the cadavers would additionally contain chemicals induced by parasitism, such as the lethal toxins generated by the endosymbiotic bacteria harbored by SC. Uninfected beetle cadavers were produced by euthanizing adult beetles in a freezer at −20 °C for 24 h—a common approach for avoidance tests with insects (eg Maák et al. 2020).

BB-infected cadavers were produced by exposing individual beetles to 700 µL of a 10^8^ conidia/mL solution of BB in a Petri dish lined with 90 mm grade 1 Whatman filter paper. Beetles were observed daily, and after death had occurred, the cadavers were placed on damp filter paper and incubated at 25 °C. These BB-killed cadavers were used in the behavioral trials after incubating for 14 d, at which point fungal growth was visibly emerging from the cadaver (ie at the sporulating stage). We used this approach rather than incubating BB on freeze-killed corpses, as per Maák et al. (2020), to mimic natural infection processes as closely as possible. To produce SC-infected cadavers, beetles were exposed in groups of 10 to 700 µL of a solution containing 1 IJ/mL in a Petri dish lined with filter paper. Beetles were observed daily for death, at which point they were kept damp and incubated at 25 °C. SC-killed cadavers were used 9 d after death as previous studies have indicated that IJ emergence occurs after 11 d, and other investigations of avoidance used cadavers that were 2 d away from nematode emergence (eg Grunseich et al. 2021). Infection in the SC-killed cadavers was confirmed using dissection after the behavioral trials were completed. Owing to this infection confirmation step for SC, and the visible fungal growth on BB-infected cadavers, all parasite-killed cadavers were considered to be infected.

### Choice tests

**Figure 1:**
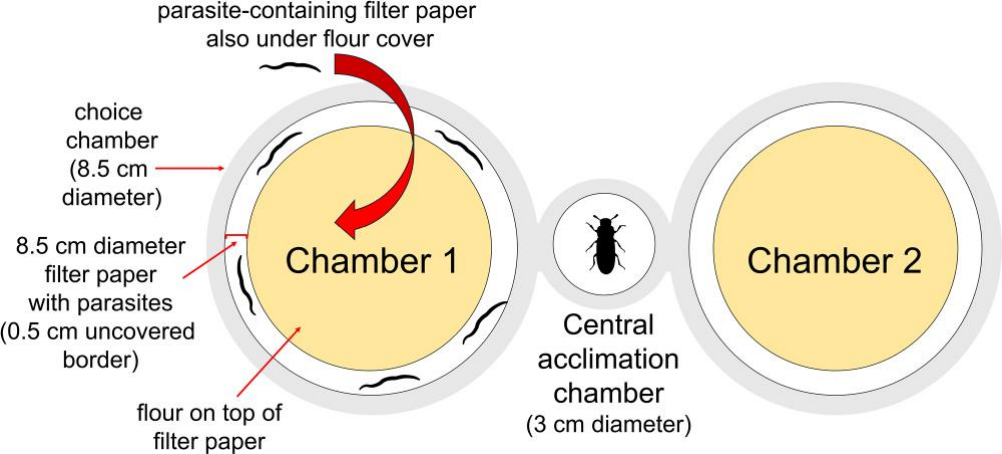
Schematic of choice arena set-up for behavioral experiments described in Table 1. The design for choice test B (presence of the nematode SC vs. no threat) is provided as an example. Note that the beetle is not to scale.

We used choice arenas (Carolina Biological) to investigate beetle avoidance of cues indicating parasite infection risk. The arenas comprised 3 circular chambers; the 2 larger chambers on both ends had an internal diameter of ∼8.5 cm, and a smaller middle chamber had a diameter of 3 cm for a total internal length of 20 cm and an internal height of 1.5 cm (see Fig. 1 for arena set-up). All the chambers were lined with 85 mm grade 1 Whatman filter papers during the choice tests. The conditions for all choice tests are listed in Table 1, but these were flipped for half of the replicates during the trials for each to address the possibility of directional bias by the beetles despite pilot behavioral trials indicating no inherent preference by beetles for 1 side of the choice arena over the other when only flour was present in both chambers.

For the single-threat trials (choice tests A to E and K in Table 1), the filter paper in either chamber contained an experimental condition based on its assigned treatment. Pathogen threat was represented by a solution of BB conidia (10^7^ conidia/mL) or 1 containing SC (1 IJ/µL) directly added to the filter paper before flour was later added (see below). Our preliminary infection experiments had shown these concentrations to be lethal to the beetles, and we opted to inoculate the filter paper so the flour was kept dry, stopping it from becoming adhesive. For the no threat condition in all choice tests, the filter paper was dampened with either 0.05% Tween 80 or DW when the choice test involved comparison to BB or SC infectious stages, respectively. In the conditions with solutions containing a dual parasite threat (both BB and SC infectious stages), the Tween 80 concentration in the parasite solution was half that of the BB-containing solution given that the SC-only solutions did not involve Tween 80. Pilot behavioral trials also indicated that beetles did not avoid chambers containing filter paper dampened with 0.05% Tween 80 more than chambers holding filter paper dampened with DW (F_1,28_ = 0.855, *P* = 0.363).

The single-threat trials also involved a choice between a EC and a negative control (choice test K) as the euthanized cadaver should represent a general danger cue. To do this, euthanized cadavers were defrosted for an hour before trials to ensure they were fully thawed before use. Afterward, the cadavers were placed on the edge of the flour pile closest to the middle, approximately 1.5 cm away from the center of the arena. In trials that contained parasite infectious stages plus cadavers (ie the mixed cues choice tests represented by G to I and M to O in Table 1), euthanized cadavers were defrosted and placed in the same position described above. Defrosting of parasite-killed cadavers was not necessary given that they were killed by their respective parasite instead of freezing, and they were also positioned in the same way as the euthanized cadavers. BB or SC infectious stages were added to the conditions at the same concentrations and using the same methods as described for the single-threat tests above.

After creating the experimental conditions in the choice chambers that corresponded to the focal choice test, the arenas were allowed to dry for 20 min before 5 g of flour media (the same used for beetle culture) was added to both choice chambers, so it covered the majority of the filter paper, leaving a ∼0.5 cm uncovered border over which the beetles needed to pass to access the flour (Fig. 1). This not only permitted physical contact between the beetles and the parasite-inoculated filter papers but also allowed any chemical cues to permeate into the air of the chamber. A plastic cone ∼3 cm tall with an internal diameter of 1.5 cm was placed in the center chamber of each arena to contain a haphazardly selected beetle that was left to acclimate for 5 min until the trial began. Experimental trials were started by removing the cone, giving the beetle access to both choice chambers. Once the trial commenced, the beetles were recorded using digital cameras (2 JVC HD 203 Everio digital and 1 Canon VIXIA HF R700) under red light as they cannot see this wavelength (Duehl et al. 2011), allowing them to remain in darkness and encourage movement while providing sufficient light to record their position. Beetle behavior was recorded for 30 min as pilot behavioral trials showed this to be sufficient for the beetles to explore the arena and make a choice. Trials were conducted over 15 recording days and split as evenly as possible within the following clusters (see Table 1): single threat (choice tests A, B, and K), dual parasite threat (tests C, D, and E), and mixed cues involving either SC infectious stages in combination with euthanized or SC-killed cadavers (tests F to J) or BB infectious stages in combination with euthanized or BB-killed cadavers (tests L to P). Between trials, the arenas were washed with hot soapy water and then wiped down with ethanol before being left to dry completely. Beetles were not reused and were euthanized at the end of each trial; each replicate for all trials and choice tests thus consisted of unique individual beetles.

### Behavioral and statistical analysis

Video recordings were analyzed by noting the position of the beetle every 30 s as either in the left, right, or center chamber of the arena, as well as whether they were actively moving. We then calculated the proportion of time spent by the beetle in each arena section. For each choice test, we considered whether the data was skewed (Kurtosis value greater than ±1) and performed an arcsine square root transformation if so to meet the assumption of an underlying normal distribution. The proportion of time that each beetle spent in the chamber representing the anticipated high-risk condition (see Table 1) was analyzed separately for each choice test using a generalized linear mixed model (GLMM) procedure with a normal distribution and identity link function. We used separate GLMMs because the high-risk condition was not consistent among the choice tests (Table 1). Beetle identity, the chamber containing the high-risk condition, and the day of the trial were included as random effects.

To account for the testing of multiple independent hypotheses, we applied the Benjamini–Hochberg procedure with a false discovery rate of 0.25 to determine which of the *P* values resulting from the separate GLMMs were significant. However, this was applied separately to each cluster of choice tests (ie parasite-only cues, SC mixed cues, and BB mixed cues) as each cluster represented a distinct theme within which overall comparisons could be made. As such, all choice test output below and in Table 1 reflects significant *P* values based on the Benjamini–Hochberg procedure. We included the choice test involving a euthanized cadaver versus no threat (choice test K) in the parasite-only cues cluster rather than arbitrarily including it in either of the mixed cue clusters. Statistical analysis was conducted using the IBM SPSS version 29.0.1.0.

## Results

### Parasite lethality

After exposure to IJs of SC during the pilot trials, 20% of *T. castaneum* died within 4 d, but no mortality was seen in the control beetles at that point. Beetles exposed to BB conidia showed a 50% mortality rate after 11 d, with a 10% death rate in the controls. These mortality rates indicate that the doses of BB and SC to which the beetles were exposed in the choice tests presented a serious threat that they could have theoretically tried to avoid.

### Parasite-only cues

**Figure 2:**
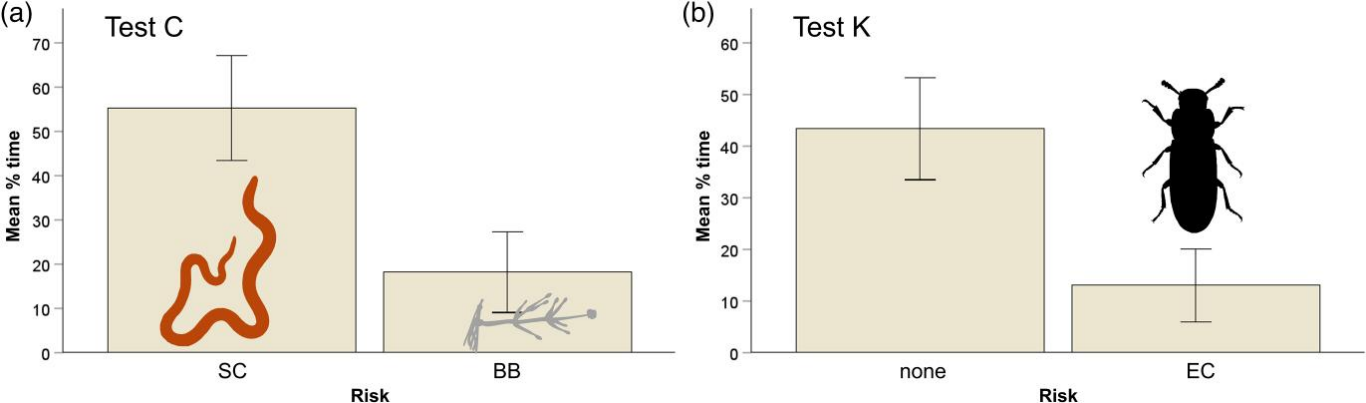
Mean (+ S.E.) proportion of time spent by red flour beetles (*T. castaneum*) when given a choice between the following single cue threats: a) SC infectious stages versus BB infectious stages, and b) no threat versus a euthanized cadaver. See Table 1 for lettering corresponding to the choice tests.

The results of the individual GLMMs for this cluster of choice tests (A to E and K) indicated that the beetles showed no significant avoidance of chambers containing single threats posed by the infectious stages of BB or SC. When choosing between BB and no threat (test A), beetles did not avoid BB (*P* = 0.569—see Table 1 for full statistical output; Fig. 2). The same was true when choosing between SC and no threat (test B; *P* = 0.596). However, when the beetles had to choose between the 2 sides of the arena containing either SC or BB (test C), they showed significant avoidance of BB (*P* = 0.02). When choosing between a chamber containing a dual threat of both BB and SC versus only 1 of these parasites, the beetles did not avoid the dual threat versus BB alone (test D; *P* = 0.755), nor did they avoid the dual threat when the other chamber had only SC (test E; *P* = 0.276). When choosing between chambers containing either a EC cadaver or no threat (test K; *P* = 0.008), the beetles showed significant avoidance of the cadaver.

### Parasite and conspecific cues (mixed)

**Figure 3:**
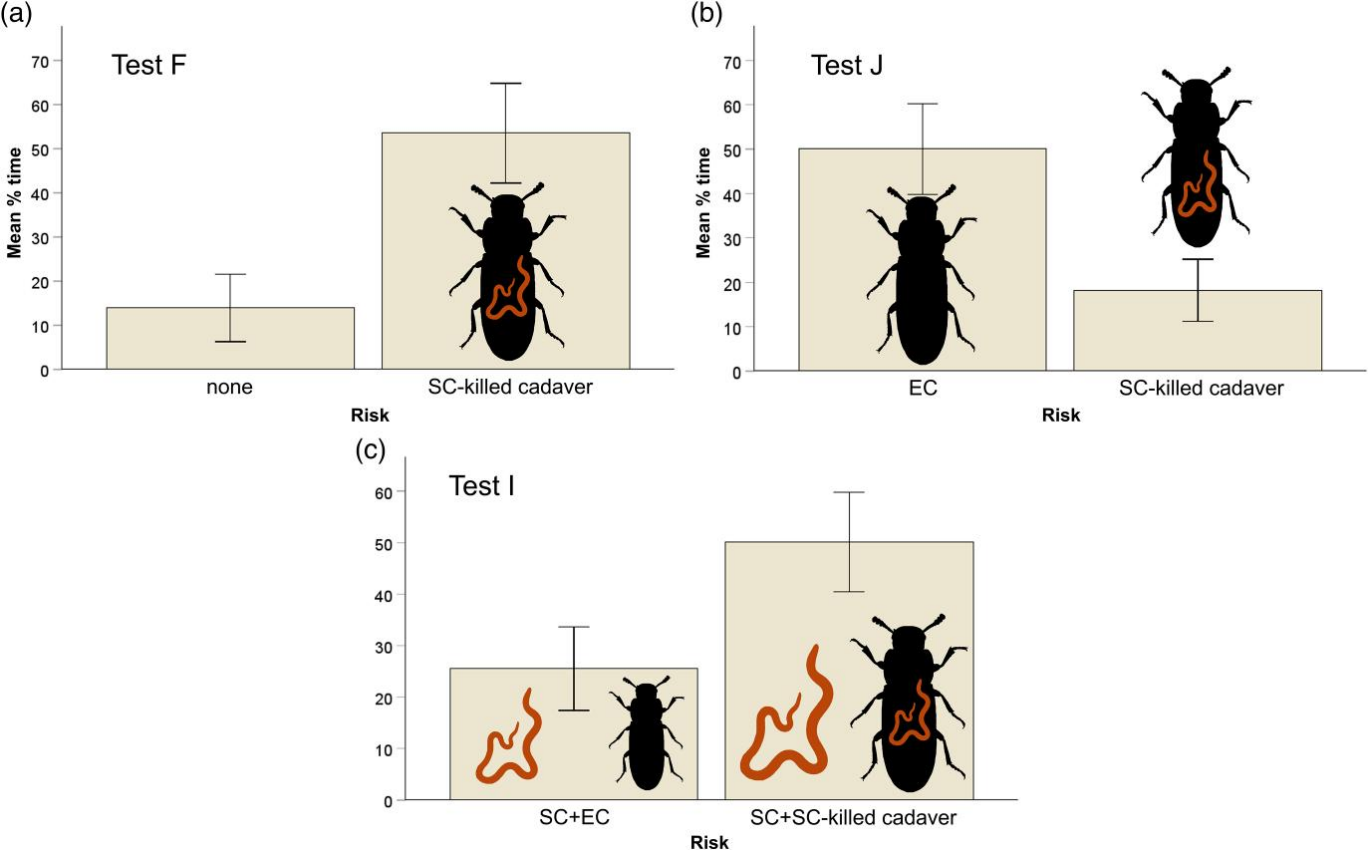
Mean (± SE) proportion of time spent by red flour beetles (*T. castaneum*) when given a choice between the following mixed cue threats: a) no threat versus an SC-killed cadaver, b) a euthanized cadaver versus an SC-killed cadaver, and c) SC infectious stages and a euthanized cadaver versus SC infectious stages and an SC-killed cadaver. See Table 1 for lettering corresponding to the choice tests.

When choosing between chambers with uncontaminated flour (no threat) and uncontaminated flour with an SC-killed conspecific (test F), the beetles spent significantly more time on the side with the SC-killed cadaver (*P* = 0.006; Fig. 3). However, the beetles showed no avoidance when given the option between SC with a euthanized cadaver and SC alone (test G; *P* = 0.275). Similarly, there was no difference between the condition containing a euthanized cadaver and SC with a euthanized cadaver (test H; *P* = 0.625). However, when the options were SC with a euthanized cadaver or SC with an SC-killed cadaver (test I), the beetles spent significantly more time around the latter (*P* = 0.057). In contrast, SC-killed cadavers on their own were avoided when the alternative was uncontaminated flour with a euthanized cadaver (test J; *P* = 0.017).

For all choices involving BB in combination with conspecific cues, beetles showed no avoidance of either arena chamber regardless of the condition (low or high risk) it contained. They did not avoid chambers containing uncontaminated flour with a BB-killed cadaver when the other had uncontaminated flour (test L; *P* = 0.06), nor was there greater avoidance of chambers with BB and a BB-killed cadaver over chambers with BB and a euthanized cadaver (test M; *P* = 0.587). There was also no significant difference in chamber choice when the option was uncontaminated flour with a euthanized cadaver versus BB with a euthanized cadaver (test N; *P* = 0.978), and when the choice was between BB with a euthanized cadaver and BB with a BB-killed cadaver (test O; *P* = 0.902). Lastly, beetles exhibited no difference when the choices were uncontaminated flour with a euthanized cadaver and uncontaminated flour with a BB-killed cadaver (test *P*; *P* = 0.343).

## Discussion

Overall, red flour beetles (*T. castaneum*) did not exhibit antiparasite behaviors in the form of avoidance, either in response to parasite-only cues from infectious stages of the fungus BB or the nematode SC, or to cues associated with dead conspecifics that had died of infection. The beetles also did not generally show greater avoidance of situations containing multiple threat cues from combinations involving both BB and SC and/or cadavers, suggesting that they do not gauge or respond to parasitism risk in this way. Rather, the beetles seemed to be attracted to SC-killed cadavers.

Despite the lack of overall parasite avoidance behavior, there were some instances where this was observed. For instance, while the beetles did not choose parasite-free chambers when the other option was the single threat of either BB or SC infectious stages, they opted for chambers with SC when the alternative was BB. When chambers contained a mix of SC and cadavers, beetles avoided these if the cadaver had died of SC infection compared to a EC; however, SC-killed cadavers often seemed to have an attractive effect on *T. castaneum*. This was not seen for BB-killed cadavers. Our results from test K also confirm that euthanized cadavers had a repellent effect on beetles when no other cues are present, consistent with previous studies (Trematerra et al. 1996).

As noted above, we did not observe *T. castaneum* avoidance of BB fungal spores (conidia) in the choice trials—this contrasts with the findings of Ghayeb et al. (2019), who saw red flour beetles avoid grains contaminated with BB conidia. In addition, other insects, including various ant and beetle species, have been reported to avoid the conidia of BB and other entomopathogenic fungi (Ormond et al. 2011; Tranter et al. 2015; Dotaona et al. 2017), although some are attracted to volatiles emitted by BB (eg Geedi et al. 2023). Similarly, we observed neither avoidance of nor attraction to BB-killed cadavers. While this is generally in keeping with results from previous studies involving BB or other entomopathogenic fungi, there is considerable variation as sometimes avoidance is seen (eg Rännbäck et al. 2015; Janmaat et al. 2022) and sometimes it is not (eg Pontieri et al. 2014).

There are various possible explanations for why our red flour beetles did not exhibit strong avoidance of BB infectious stages or via BB-killed corpses. One reason is beetles’ ability to perceive cues pertaining to fungal risk. We covered the filter papers containing BB and/or cadavers with flour to encourage beetle movement, but if contact chemoreception plays a role, as seen for tick attraction to BB-inoculated substrate (Gooding et al. 2024), this may have been impeded despite the uncovered filter paper border over which the beetles had to cross to reach the flour. The substrate may also have required a longer incubation time to elicit an effect (Gooding et al. 2024). In addition, insect responses to conidia can vary with substrate type based on the preferred substrate of the insect species (Meyling and Pell 2006).

While we observed a relatively high mortality rate, the fitness cost(s) of infection can vary by host sex and life stage such that not all might exhibit parasite avoidance. For instance, adult males would have time to mate after BB infection as it typically takes many days for the fungus to begin killing its host (50% lethality after 11 d here). Their fitness costs could thus be much lower than those to females, which produce and lay hundreds of eggs over a span of >300 d in red flour beetles (Good 1933), such that they would likely have greater loss of reproductive output if infected. Larval stages would also suffer great fitness losses as they would lose their opportunity to reproduce altogether. The influences of life history in this context thus require further study and future investigations that specifically consider differences in BB avoidance based on sex and developmental stage would be helpful.

Another possible explanation for why red flour beetles did not generally avoid BB threat in our study is that they instead rely more on other behavioral defenses, such as increased grooming, a common strategy exhibited by many hosts when they come into contact with an infectious stage or ectoparasite that must attach and penetrate (Hart 1994; Mooring et al. 2004). Notably, various ant and beetle species exhibit increase grooming when exposed to pathogenic fungal conidia (Tranter et al. 2015; Nuotclà et al. 2019)—this should be explored with red flour beetles. Lastly, different strains of BB also have different degrees of repellent effect (Mitina et al. 2020)—also an area for future study in terms of host avoidance behaviors.

In addition to employing other behavioral defenses, such as grooming, as an alternative to parasite avoidance, the beetles may have been relying on chemical defenses. Specifically, many arthropods, including beetles, such as *T. castaneum*, secrete quinone compounds (eg Rocha et al. 2013; Pedrini et al. 2015). These quinone secretions have known antimicrobial effects, including a well-demonstrated ability to inhibit BB (eg Yezerski et al. 2007; Pedrini et al. 2015; Smith and Koprivnikar 2024). Given the effectiveness of quinone secretions at inhibiting BB growth during acute exposure periods of <24 h (Smith and Koprivnikar 2024), beetles may not try to avoid BB if their chemical defenses are sufficient in the short term, especially if there are NCEs, such as missed foraging opportunities. However, chronic BB threat may elicit avoidance behaviors and should be investigated given that quinone secretions lose their effectiveness over time (Smith and Koprivnikar 2024).

For the 1 test where BB was avoided, the alternative was SC. Considering the outcome of host contact, we expected that the more dangerous threat (BB here based on lethality data) would be more avoided, which is what we observed. However, it is not evident why the beetles would avoid BB in this situation but not when the alternative was a parasite-free area. This said, it is possible that inconsistent BB threat avoidance was not just driven by risk, but also by detectability. BB reproduces by dispersing conidia through the air, also producing airborne VOCs that can be detected by insects (Jalinas et al. 2022). Because nematodes do not have airborne stages, the spatial distribution of their detectible VOCs (eg Grunseich et al. 2021) could be smaller, making it easier for our beetles to detect the threat represented by BB compared to SC. However, if avoidance of BB was consistent owing to ease of detection, we should have seen this in the single-threat condition, or even possibly when it was present in the dual threat conditions, but did not. This suggests a role for the other potential contributing factors discussed above.

Our results indicate no overall avoidance of SC IJs by *T. castaneum* and represent the first study to consider avoidance as a behavioral defense for this host–parasite system. As a whole, there have been very few investigations of avoidance behavior against entomopathogenic nematodes in other insects, especially any considering IJs, but some instances have been reported (eg Ennis et al. 2010). Importantly, just as for pathogenic fungi, grooming is an effective behavioral defense by insects against nematodes. Grooming removes nematodes from the body of the host before they can penetrate and establish an infection (Mankowski et al. 2005) and has been seen in various beetles (eg Gaugler et al. 1994; Ennis et al. 2010). It is thus possible that *T. castaneum* instead engaged in other behavioral defenses, such as increased grooming activity, which were not captured here but are an area for future work.

An additional factor is the host-finding strategy and associated activity level for different EPNs. Importantly, Grunseich et al. (2021) showed that cucumber beetles avoid EPNs that “cruise” for hosts but not “ambushers,” such as SC, who wait for these to come near, perhaps because the latter have been selected for olfactory crypsis as part of their ambushing strategy but also because the EPNs have different bacterial symbionts. However, differences in EPN activity could also affect the quantity of any airborne cues generated. Finally, insect quinone secretions are not just harmful to fungi, such as BB, but to SC as well (Smith et al. 2023). This further reinforces the possibility that these beetles rely on chemical defenses over avoidance for many types of parasites, explaining why they did not generally avoid either BB or SC here despite the threat posed by both parasites. The general effectiveness of quinones in this context could explain why beetles and other arthropods invest substantial resources into these secretions, which require both specialized cuticle-lined storage glands and constant secretion (Happ 1968; Pedrini et al. 2015). With this investment into chemical defense, perhaps the additional costs potentially associated with parasite avoidance make it less likely outside of particular circumstances.

A key reason why red flour beetles may not use avoidance as a defense against parasites could be due to the associated NCEs. As other studies of insect–parasite systems have shown, avoiding parasites can be expensive, including metabolic costs and reduced foraging output (eg Orr 1992; Luong et al. 2017). Considering the demonstrated and potential NCEs associated with entomopathogens, it is possible that *T. castaneum* did not engage in parasite avoidance here for this reason; ie the cost(s) were too high. Notably, red flour beetles are ground foragers, primarily feeding on damaged grains (Trematerra et al. 2015), which may also influence the cost(s) of avoiding parasitic nematodes and fungi found in/on the soil compared to other defenses, such as chemical secretions. While not studied in insects, several studies with birds have shown that ground foraging is associated with greater risk of parasite exposure and yet they can defend themselves against infection (eg Dolnik et al. 2010; Dumas et al. 2022). If parasite avoidance limits red flour beetle foraging opportunities, they may also rely more on other defenses, such as quinone secretions or grooming. Studies using other insects with similar foraging strategies are needed to establish the broad occurrence of parasite avoidance, as well as key influences, such as food availability (eg Kabaluk and Ericsson 2007). It is possible that red flour beetles also engage in parasite avoidance, but only under specific circumstances (eg life stage and food availability), which warrant study. Parasite avoidance may only manifest at different spatial scales (Love et al. 2024); thus, studies considering this aspect will also be important.

Not only did *T. castaneum* fail to avoid conditions containing infective stages of BB or SC, but our results also indicate the beetles were generally attracted to, rather than repelled by, conspecifics killed by SC as they spent more time in chambers containing SC-killed cadavers in 2 of the 3 tests with this choice. As noted above, avoidance of EPN-infected cadavers varies depending on the species of nematode involved, likely due to their distinct VOC profiles (Grunseich et al. 2021). Interestingly, when analyzing the VOCs released from cadavers infected with 1 of 2 *Steinernema* species (including SC), Li et al. (2024) found the presence of pentadecane, which is also found in *T. castaneum*-infested flour (Niu et al. 2016). This might help explain some of the attraction toward SC-killed cadavers here as pentadecane is known to attract other herbivorous insects (eg Karmakar et al. 2016; Mitra et al. 2020). However, further study is needed to determine if this contributed to our results.

This said, red flour beetle attraction to SC-killed cadavers here was not constant. In the absence of SC IJs in either choice chamber, beetles spent significantly less time near SC-killed cadavers compared to euthanized cadavers. As we found avoidance of euthanized cadavers when the alternative was a chamber with no cues, consistent with previous reports that conspecific cadavers are a repellent in *T. castaneum* (eg Trematerra et al. 1996), this discrepancy regarding an apparent attraction to conspecifics killed by SC, but not BB, in some of the choice tests may be due to various factors. One possible reason could involve infection intensity. We considered cadavers as only infected or uninfected, not quantifying the number of nematodes in each. However, intensity of infection may influence the avoidance of conspecific cadavers as seen with other insect–pathogen systems (eg Maák et al. 2020), and cadavers with fewer nematodes may have been less attractive to the beetles, especially if this potentially affected their VOC profiles—this should be explored in future work, as well as beetle avoidance of SC- versus BB-killed cadavers.

While we did not explore potential mechanisms of beetle attraction or repellence to different cadaver types, these likely involve VOCs that act as alarm cues to signal danger to conspecifics. For instance, 1-pentadecene is secreted by various insects, including red flour beetles, and can repel conspecifics (Suzuki et al. 1975). Fatty acid “death cues” are also known to play a role in cadaver avoidance or attraction in other insect species (eg Sun et al. 2013). However, 1-pentadecence acts as an attractant in low concentrations and as a repellent only as its concentration increases given its role in managing beetle population densities (Đukić et al. 2021). Interestingly, in the choice tests involving 1 chamber with a euthanized cadaver but BB conidia present in both sides, cadaver avoidance was not seen. This could indicate that BB infectious stages themselves emit VOCs that mask chemicals normally triggering avoidance in beetles. Further research into this aspect would be helpful, including conditions that consider infection intensity and cadaver status (frozen vs. fresh), as well as VOC profiles.

Because SC-killed cadavers were not avoided by beetles here, this also suggests the potential for attractant VOCs, such as 1-pentadecene (Suzuki et al. 1975), especially if the high degree of physical damage to cadavers caused by SC infection versus intact ECs resulted in attractant and not repellent levels. It is also possible that beetle attraction to SC-killed cadavers in many of our choice tests could be an adaptive manipulation by SC to increase the odds of transmission. Such strategies have been seen for other parasites using insect hosts. For instance, fungi can mimic female sex pheromones to attract male flies (Naundrup et al. 2022), and *Tribolium confusum* showed a greater preference for rat feces containing tapeworm eggs over uninfected feces due to attractant volatiles (Evans et al. 1998). However, further research would be needed to identify potential mechanisms and demonstrate such benefits to SC.

To conclude, it does not appear that avoidance behavior is a primary defense against BB and SC by *T. castaneum*, but further research is needed to contextualize parasite avoidance by this insect as this can depend on biological sex or life stage. Investigating how these factors influence avoidance would be a logical next step, as well as considering potential mechanisms (eg VOCs). This is particularly relevant for the use of entomopathogens in flour beetle biocontrol given the implications for food safety and human health. If pest insects are able avert contact with parasite infectious stages or infection-killed conspecifics, thus lowering the chances of successful infection and the efficacy of biocontrol measures, potential strategies to counter parasite threat avoidance should be explored. It is also important to consider how exposure to concurrent threats can influence avoidance, such as those posed by predators and parasites as well as different types of parasites, and to examine avoidance with a range of insects and entomopathogens. Investigating parasite avoidance is critical for understanding the contribution(s) of host behaviors to their general defense strategies, especially for host taxa, such as insects, where this has been relatively less studied, and the growing urgency to identify the overall impacts of parasites on hosts through both their CEs and NCEs.

## Data Availability

Analyses reported in this article can be reproduced using the data provided by Smith and Koprivnikar (2026).
